# Natural Aging Life Prediction of Rubber Products Using Artificial Bee Colony Algorithm to Identify Acceleration Factor

**DOI:** 10.3390/polym14173439

**Published:** 2022-08-23

**Authors:** Xiaohui Guo, Xiaojing Yuan, Genliang Hou, Ze Zhang, Guangyong Liu

**Affiliations:** 1 Combat Support College, Rocket Force University of Engineering, Xi’an 710025, China; 2 Key Laboratory of Rubber and Plastic Materials and Engineering, Ministry of Education, Qingdao University of Science and Technology, Qingdao 266042, China

**Keywords:** artificial bee colony, rubber aging, acceleration factor, life expectancy, improved Arrhenius equation

## Abstract

We aim to predict the natural aging life of 8016 ethylene propylene rubber accurately and quickly. Based on the time-temperature equivalent superposition principle, the artificial bee colony algorithm was introduced to calculate the acceleration factor of the accelerated aging test, and the calculation of the acceleration factor was considered an optimization problem, which avoided the error superposition problem caused by data fitting at each temperature. Based on the traditional Arrhenius equation, a power exponential factor was introduced to consider the non-Arrhenius phenomenon during the rubber aging process. Finally, the aging prediction curve of 8106 ethylene propylene rubber at 25 °C was obtained. The prediction results show that the artificial bee colony algorithm can quickly and accurately identify the acceleration factor of the accelerated aging test. The dispersion coefficients between the predicted and measured results of the improved and traditional Arrhenius equations are 1.0351 and 1.6653, respectively, which indicates that the improved Arrhenius equation is more advantageous in predicting the long-term aging process of rubber products.

## 1. Introduction

Rubber is a typical engineering material widely used in tires, air springs, and various sealing elements. Rubber experiences aging as a polymer material, with a gradual decline in properties until complete failure occurs under the action of heat, oxygen, light, mechanical stress, and other factors [1,2]. If rubber products are not replaced before failure, this can cause equipment failure or accidents. Therefore, the accurate prediction of the lifetimes of rubber products has important theoretical significance and engineering value. Accurate and efficient accelerated aging test methods and corresponding data-processing methods are being widely studied for accurate reliability assessments and prediction of the degradation laws of rubber products in use environments [3,4,5,6].

The lifetimes of rubber products can be evaluated using both natural and accelerated aging tests. Because rubber product lifetimes range from 3 to 25 years, depending on the rubber material used, natural aging tests can be time-consuming and are unsuitable for the product design stage. Based on the time-temperature equivalence principle, which states that the rate of the rubber aging reaction increases with increasing temperature and the time until failure is significantly reduced, accelerated aging tests are widely used in the lifetime prediction of rubber products. By identifying the acceleration factors at various high temperatures, the accelerated aging test extrapolates these acceleration factors to the use environment of the product and then predicts its failure lifetime [7]. The accuracy of the high-temperature acceleration factor identification affects the results of the acceleration factor extrapolation in the use environment, which in turn affects the accuracy of the lifetime prediction. Therefore, accurately identifying the high-temperature acceleration factors is necessary for accelerated aging tests, as this identification directly determines the accuracy of lifetime prediction [8,9,10]. 

Each acceleration factor is generally calculated using the rate of the aging reaction or pseudo-life at a given aging temperature. By fitting the decay data of the aging performance indexes at different aging temperatures, the time when the rubber reaches the failure threshold at each aging temperature, that is, the pseudo-life at that temperature, or the rate of the aging reaction at that temperature, is predicted. The ratio of the pseudo-life or the aging reaction rate at each aging temperature to that at the reference temperature is the acceleration factor. The mathematical model of the aging performance index decline trajectory significantly affects the final result because this method must fit the data at all aging temperatures, and the superposition of errors at each aging temperature seriously hinders the determination of an accurate acceleration factor. Emerging intelligent algorithms can effectively avoid the error superposition problem caused by multiple fittings and have a high application value in acceleration factor identification.

In terms of accelerated aging data processing, traditional data processing methods need to fit the aging data at each temperature multiple times, which will cause the superposition of errors and the efficiency is too low. In this paper, an artificial bee colony algorithm is used to determine the optimal value of the acceleration factor for accelerated aging tests, and the proposed method encodes the acceleration factor at each aging temperature as a candidate solution, which can simultaneously improve the accuracy and speed of accelerated aging data processing. Considering the non-Arrhenius phenomenon in the aging process, a power exponent factor was introduced into the traditional Arrhenius equation, and the aging life of 8106 ethylene-propylene rubber in the natural environment was finally predicted based on the time-temperature equivalent superposition principle. To verify the accuracy of the improved Arrhenius equation in predicting the aging life of rubber products, the improved and traditional Arrhenius equation life prediction results were compared. The results show that the improved Arrhenius gives more accurate predictions during the long-term aging of rubber products.

## 2. Accelerated Aging Test Model and Principle

### 2.1. Constant-Stress Accelerated Aging Test

The life span of rubber products in natural environments is approximately 3–25 years or even longer, which hinders the study of their reliability and life span. Accelerated aging tests based on the principle of time–temperature equivalence can achieve rubber aging in a short period and predict the actual service lifetimes of rubber products by processing data obtained at multiple aging temperatures. At present, the accelerated aging test is the most common method for determining the service lifetimes of rubber products [11]. Accelerated aging tests are performed via three methods: constant stress, step (decreasing) stress, and sequential stress. The step (decreasing) stress accelerated aging test requires the least time, but the data processing method is not sufficiently mature, so constant-stress accelerated aging is used.

The rubber studied in this paper was 8106 ethylene propylene rubber, which is commonly used in the sealing structures of launch vehicles. According to the working principle of the sealing components, the permanent deformation rate under compression relates directly to the sealing performance and reflects the working performance of the sealing components; therefore, the permanent deformation rate under compression is chosen as the performance decline index of the accelerated aging test. The rubber compression set specimens are processed according to ISO 895:1991 and placed in the corresponding test fixtures, applying the same stress conditions to the compression set specimens as they would actually work.

In the preparation stage of the test, the specified compressive stress is applied to the rubber. After being placed at room temperature for 24 h, the clamp is loosened, and the sample is placed in a free state for 1 h to measure its recovery height before aging and continue to compress to the specified deformation rate for the aging test.

The accelerated aging test is carried out according to GJB 92.1-1986. The tester was installed in the aging test box, and the aging temperature was set to 80 °C, 90 °C, 100 °C, and 110 °C, respectively. The aging temperature was lower than the maximum temperature that 8106 ethylene–propylene rubber can be used to ensure that its aging mechanism is not changed. After reaching the specified aging time, take the test tool out of the aging box, cool it at (25 ± 1) °C for 2 h, loosen the clamp, let the sample stand in a free state for 1 h, measure the recovery height after compression, calculate compression set retention. Three samples were measured at each time point, and the median of the three measurements was taken as the final measurement.

### 2.2. Principle of Time-Temperature Equivalent Superposition

The principle of time–temperature equivalence means that the degradation of the properties of rubber caused by aging can be observed at different temperatures; therefore, the aging failure of rubber can be accelerated by high temperatures, thus allowing extrapolation of its lifetime under natural conditions or service environments [12]. Numerous studies have shown that the aging reaction rate of rubber at different temperatures follows the Arrhenius equation [13,14,15]:

(1) $$k(T)=Ae^{-\frac{E_{a}}{RT}}$$

where k(T) represents the aging reaction rate at the aging temperature of T; A is a temperature-independent constant; $E_{a}$ is the apparent activation energy of the aging reaction R is the ideal gas constant.

Both sides of Equation (1) can be linearized by taking logarithms simultaneously:

(2) ln(k)=a+b/T

Comparing Equation (2) with Equation (1) yields the apparent activation energy as follows:

(3) $$E_{a}=-bR$$

Recent studies have shown that the activation energy of rubber materials is not constant in aging at different temperatures; this is called a non-Arrhenius phenomenon [16]. The Arrhenius equation can be used to predict the aging life of rubber with certain deviations [17,18]. Therefore, an improved Arrhenius equation was proposed [19]:

(4) $$k(T)=Ae^{-{\left(\frac{E_{b}}{RT}\right)}^{n}}$$

Here, $E_{b}$ is the actual activation energy of the aging reaction, n is a power exponential factor independent of temperature, and the rest of the parameters have the same meaning as in Equation (1). The logarithmic transformation of Equation (4) yields the following form:

(5) $$ln(k)=a+b/T^{n}$$

The slope of the curve Ln(k)−1/T can be obtained by deriving Equation (5) from 1/T and multiplying this slope by −R to yield the equivalent linear activation energy:

(6) $$E_{a}=-nbR/T^{n-1}$$

Comparing Equation (3) with Equation (6), the equivalent linear activation energy obtained from the improved Arrhenius equation is a function of temperature, whereas the apparent activation energy obtained from the conventional Arrhenius equation is a constant. The improved Arrhenius equation is more consistent with the actual situation of rubber aging.

The acceleration factor is defined as the ratio of the aging reaction rate at any given aging temperature to that at the reference temperature.

(7) $$\begin{array}{cc} a_{i} & =k_{i}/k_{0}\\ & =e^{{\left(\frac{E_{b}}{R\times T_{0}}\right)}^{n}-}{}^{{\left(\frac{E_{b}}{R\times T_{i}}\right)}^{n}} \end{array}$$

where $a_{i}$ is the acceleration factor corresponding to the temperature $T_{i}$, $k_{i}$ is the aging reaction rate at $T_{i}$, and $k_{0}$ is that at the reference temperature $T_{0}$. After obtaining the acceleration factors at high temperatures, those at lower temperatures can be obtained by the extrapolation of Equation (7).

The relationship between the aging performance degradation index of rubber P and time t can be described using a binary mathematical model P−t. Given the aging temperature $T_{i}$, the aging performance degradation law of rubber is expressed by Equation (8) [20]:

(8) $$P=Ae^{-k_{i}t^{\alpha }}$$

where A,α is a time-independent constant, and k is the aging reaction rate. To facilitate the calculation, both sides of Equation (8) are linearized by simultaneously taking the logarithm:

(9) $$Ln(P)=a-k_{i}t^{\alpha }$$

By substituting Equation (7) into Equation (9), the following is obtained:

(10) $$Ln(P)=a-k_{0}{\left(\sqrt[{\alpha }]{a_{i}}t\right)}^{\alpha }$$

Therefore, when the aging time of the rubber at temperature $T_{i}$ is t, it is equivalent to aging $\sqrt[{\alpha }]{a_{i}}t$ at the reference temperature, assuming an aging time of $t_{i}$ for the temperature of $T_{i}$. The equivalent aging time at any aging temperature relative to that at the reference temperature can be obtained as follows:

(11) $$t=\sqrt[{\alpha }]{a_{i}}t_{i}$$

The test data at any aging temperature can be shifted to the reference temperature using Equation (11).

## 3. Artificial Bee Colony Algorithm

The artificial bee colony algorithm assumes the existence of a set of operations similar to certain characteristics of honeybee honey-harvesting behavior and analogizes each solution in the search space to the location of a honey source. The adaptation value of that solution is the quality of the honey source, which mimics the honeybee’s search for a quality source, thus producing a similar search for the best solution [21].

### 3.1. Algorithmic Biology Principles

The minimal model of a bee colony consists of three parts: the employed bee, observer bee, and scout bee. More precisely, one bee goes through the three different stages of employment, observation, and scouting [22]. The identity of each bee is not constant but can change at any time during the search for nectar, depending on the bee’s random behavior and the quality of the nectar it is searching for. The tasks performed by bees during the three different periods differ, as described below.

Employed bee period: The employed bee conducts a neighborhood search based on the location information of food sources in its memory. The neighborhood search process is that of the bee’s exploitation of that honey source. When the employed bee searches for a new honey source, it evaluates the quality of that source, and a new honey source of better quality than the source causes the employed bee to update the honey source information.

Observer bee period: When the employed bee finishes collecting nectar, it returns to the hive and transmits information about the food source it has obtained to the observer bee by performing a waggle dance. The observer bee selects the corresponding nectar source based on the information provided by the employed bee.

Scout bee period: When an employed bee searches the neighborhood of an existing honey source, if the quality of the honey source does not improve after a given number of searches, the employed bee gives up the source and becomes a scout bee, which randomly searches for new honey sources over a large area.

### 3.2. Algorithm Description

#### 3.2.1. Initializing the Population

The first step of the algorithm is to initialize the positions of the honey sources, each of which is a d-dimensional vector containing the parameter values to be optimized. The d-dimensional vector is denoted as $x_{i}$, where *i* = 1, 2,..., *N*, and *N* is the number of initialized solutions, that is, the number of initial honey sources. The value of each dimension of $x_{i}$ can be expressed as $x_{i}^{j}$, where *j* = 1, 2,..., *d*, and $x_{i}^{j}$ corresponds to the solution of the problem of unknown parameters. The initial solution is randomly distributed in the search space and spread throughout the search space as much as possible. The initialization of the nectar source locations is performed using Equation (12):

(12) $$x_{i}^{j}=x^{j}{}_{\min}+rand(0,1)\cdot (x^{j}{}_{\max}-x^{j}{}_{\min})$$

where $x^{j}{}_{\min}$ is the lower bound of the *j*th dimension of the solution vector, $x^{j}{}_{\max}$ is the upper limit of the *j*th dimension of the solution vector, and rand(0,1) is a random number between 0 and 1.

#### 3.2.2. Sending Employed Bees

The employed bee performs a neighborhood search near the nectar source in the memory using Equation (13) to obtain a new nectar source $v_{i}^{j}$, evaluate the adaptation value of $v_{i}^{j}$, and choose between $x_{i}^{j}$ and $v_{i}^{j}$ according to the greedy algorithm. When the adaptation value of $v_{i}^{j}$ is better than $x_{i}^{j}$, the employed bee abandons $x_{i}^{j}$ and chooses the new nectar source; in the opposite scenario, it abandons the new nectar source and retains $x_{i}^{j}$. This process can be regarded as a bee exploiting this source. The adaptation value of $v_{i}^{j}$ can be solved by transforming Equation (14) into a minimization problem.

(13) $$v_{i}^{j}=x_{i}^{j}+\varphi_{i}^{j}(x_{i}^{j}-x_{k}^{j}),\forall i\neq k,k\in rand(1,N)$$

where $\varphi_{i}^{j}$ is a random number between −1 and 1;

(14) $$fit_{i}(x_{i})=\left\{\begin{array}{l} \frac{1}{1+f(x_{i})},f(x_{i})\geq 0\\ \frac{1}{1+\left|f(x_{i})\right|},f(x_{i})<0 \end{array}\right.$$

where $f(x_{i})$ denotes the value of the objective function of the problem to be solved.

#### 3.2.3. Observer Bees Choosing Nectar Sources

After the employed bee completes a round of searches and returns to the hive, the observer bee randomly selects a corresponding nectar source based on the nectar source information provided by the employed bee. Assuming that the nectar source selected is $x_{i}$, the probability $P(x_{i})$ that $x_{i}$ is selected can be expressed by Equation (15):

(15) $$P(x_{i})=\frac{fit_{i}(x_{i})}{\sum_{i=1}^{N}fit_{i}(x_{i})}$$

At this point, the observer bee becomes the employed bee, and Section 3.2.2 is repeated.

#### 3.2.4. Scout Bees Searching for New Nectar Sources

In Section 3.2.2, when the location information of a honey source is updated after multiple search rounds, this location is judged to be caught in the local optimum. The upper limit of the number of searches is set by the algorithm user as Limit. At this point, the employed bee gives up the nectar source, records the location of the source, and becomes a scout bee. The scout bee reselects the nectar source randomly in the solution space using Equation (16). Section 3.2.2 and Section 3.2.3 are repeated until the maximum number of iterations set by the system $iter_{\max}$ is reached, and the optimal solution recorded during the process is taken as the final solution vector.

(16) $$x_{i}^{j}=\left\{\begin{array}{l} x^{j}{}_{\min}+rand(0,1)\cdot (x^{j}{}_{\max}-x^{j}{}_{\min}),trial\geq Limit\\ x_{i}^{j},trial<Limit \end{array}\right.$$

## 4. Acceleration Factor Identification Based on the Artificial Bee Colony Algorithm

### 4.1. Acceleration Factor Identification Process

The proposed method considers the acceleration factors $\left[a_{1},a_{2},\ldots ,a_{n}\right]$ at different aging temperatures as a candidate solution and determines the optimal solution using the artificial bee colony algorithm for lifetime prediction under low-temperature aging.

Assuming that the test data obtained at each aging temperature are $\left(t_{ij},P_{ij}\right)$, i is the number of aging temperatures other than the reference temperature, i=1,2,…,n−1,n, and j is the number of tests at each temperature, j=1,2,…,m−1,m. The logarithm of $P_{ij}$ is taken so that $y_{ij}=\ln(P_{ij})$ and $\left(t_{ij},P_{ij}\right)$ are transformed into $\left(t_{ij},y_{ij}\right)$, translating $\left(t_{ij},y_{ij}\right)$ to the reference temperature $T_{0}$ using Equation (11) and converting it into $\left(\sqrt[{\alpha }]{a_{i}}t_{ij},y_{ij}\right)$. Using the least-squares method, we apply Equation (10) to perform regression analysis on $\left(\sqrt[{\alpha }]{a_{i}}t_{ij},y_{ij}\right)$ and obtain $y_{ij}$ as a function of $a_{i}$. Applying the least-squares method, the estimated value of the regression coefficients minimizes the total residual sum of squares:

(17) $$f(x)=\sum_{i=1}^{n}\sum_{j=1}^{m}\left(\hat{y_{ij}}-y_{ij}\right)$$

where $x=[a_{1},a_{2},\ldots ,a_{n}]$. The problem is thus transformed into determining the optimal solution of Equation (17), and Equation (17) is used as the objective function of the artificial bee colony algorithm to obtain the optimal solution of the acceleration factors.

The specific steps of the artificial bee colony algorithm to solve the acceleration factor are detailed in Algorithm A1 in Appendix A.

### 4.2. Acceleration Factor Identification Results

**Figure 1:**
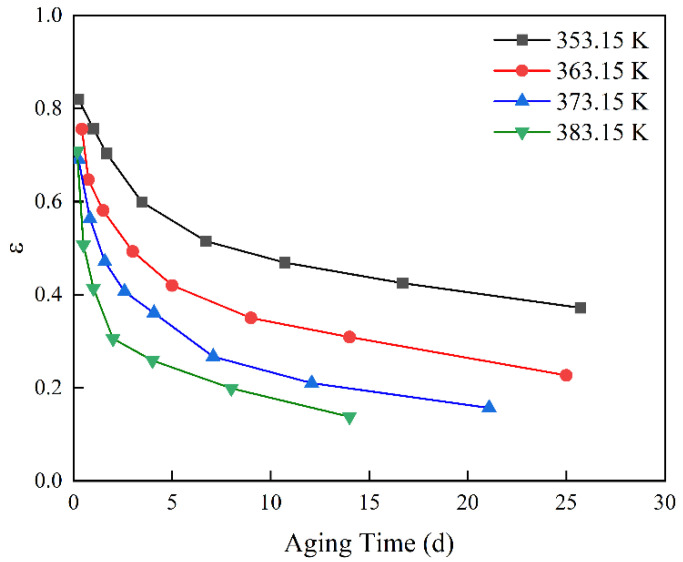
Changes in compression set retention with time.

Table 1 shows the compression set retention (ε) of 8106 ethylene propylene rubber sealing materials commonly used in launch vehicles under natural aging and constant-stress accelerated aging tests [23]. The natural aging temperature is 298.15 K, and the constant-stress accelerated aging temperatures are 353.15 K, 363.15 K, 373.15 K, and 383.15 K. Figure 1 shows the variation of compression set retention with aging time at each aging temperature, and 353.15 K is taken as the reference temperature for accelerated factor identification. The data at 353.15 K were fitted with a kinetic equation using Equation (9), and a genetic algorithm was used to identify α in the process.

**Figure 2:**
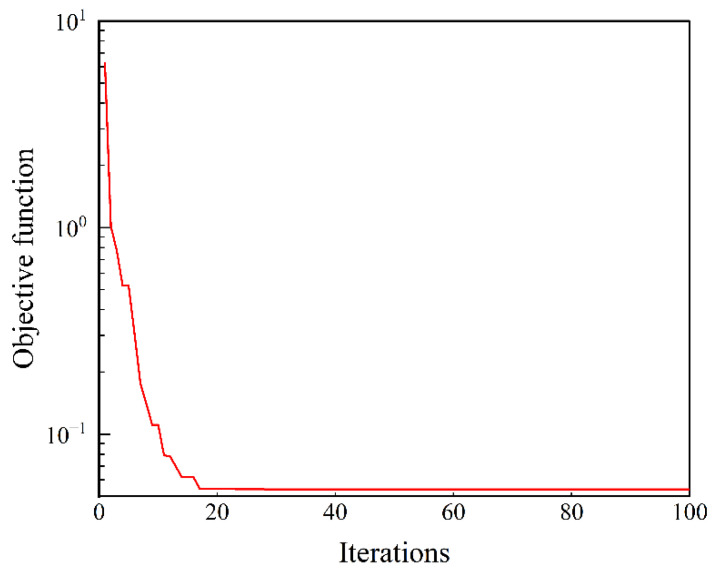
Convergence of the objective function with the number of iterations of the algorithm.

The aging data at other temperatures were translated to the reference temperature and the least-squares method was used to take Equation (17) as the objective function of the artificial bee colony algorithm. The parameters of the algorithm were set as follows: the population was set to 200, of which 100 were employed bees and 100 were scout bees, and Limit=540, $iter_{\max}=100$, $x^{j}{}_{\min}=1$, and $x^{j}{}_{\max}=10$. Figure 2 shows the convergence of the objective function value during the algorithm iteration.

**Figure 3:**
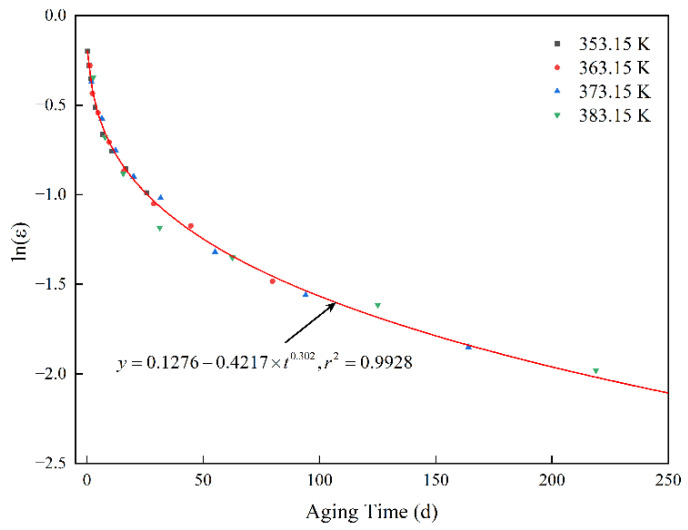
Aging data and nonlinear regression after time–temperature equivalent superposition.

Table 2 lists the identified acceleration factors obtained using the artificial bee colony algorithm. According to Equation (11), the aging data at the three temperatures of 363.15 K, 373.15 K, and 383.15 K were translated to the reference temperature, and the translated data was processed by Equation (9). Figure 3 shows the aging data after translation and the nonlinear regression curve. It can be seen that the data are distributed on the same curve. The goodness of fit of the regression curve is 0.9928, which further verifies the accuracy of the acceleration factor identification results. The regression equation is:

(18) $$y=0.1276-0.4217\times t^{0.302}$$

Combining Equations (11) and (18), the performance decay curve of the polyurethane compound at any temperature can be obtained as:

(19) $$y=0.1276-0.4217\times a_{i}t_{i}{}^{0.302}$$

## 5. Natural Aging Life Expectancy

To accurately identify the high-temperature acceleration factors, based on the least-squares method, the obtained acceleration factor is subjected to nonlinear regression analysis using Equation (7). In this process, the genetic algorithm is used to identify the parameter n as n=0.407; therefore, the regression equation is as follows:

(20) $$a=e^{{\left(\frac{7.3941\times 10^{6}}{8.314\times 353.15}\right)}^{0.407}-}{}^{{\left(\frac{7.3941\times 10^{6}}{8.314\times T}\right)}^{0.407}}$$

**Figure 4:**
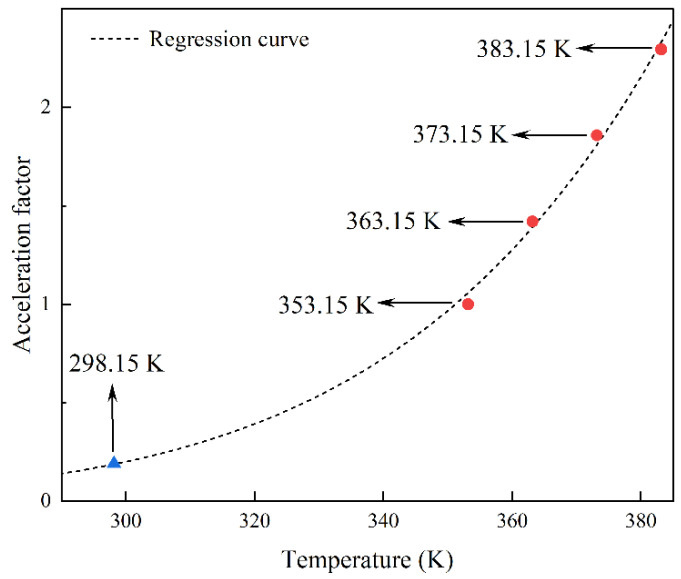
Regression and extrapolation results of the acceleration factors.

The acceleration factor at a low temperature was extrapolated using Equation (19), as shown in Figure 4. The extrapolated acceleration factor at 298.15 K is 0.1876, and it can be seen that the improved Arrhenius equation fits the acceleration factor with greater precision. To compare the accuracy of the traditional and improved Arrhenius equations in predicting rubber lifetime, the traditional Arrhenius equation was used to perform regression analysis on the obtained acceleration factors. The acceleration factors at low temperatures according to the traditional and improved Arrhenius equations are called $a_{old}$ and $a_{new}$, respectively, as shown in Table 3.

**Figure 5:**
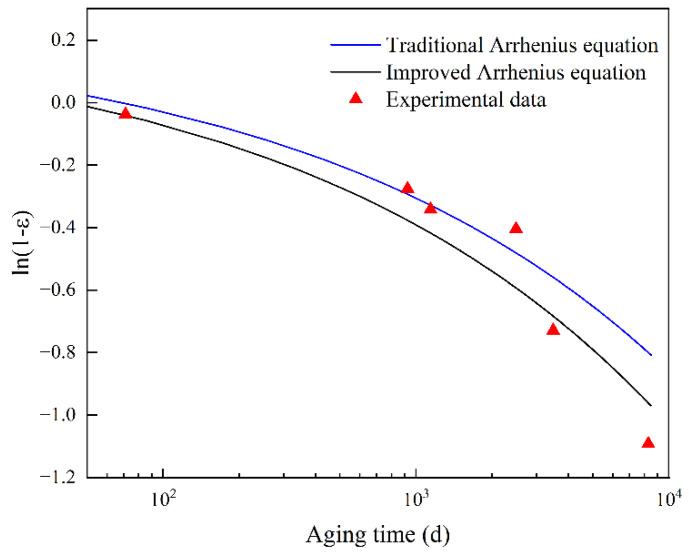
Comparison of traditional and improved Arrhenius equations in fitting experimental data.

The values $a_{old}$ and $a_{new}$ were each put into Equation (18) to obtain predicted performance degradation curves at 298.15 K, as shown in Figure 5. It can be seen that the test data are distributed around the two curves, and the two curves predict the aging performance indicators well. The prediction curve obtained by the traditional Arrhenius equation has greater prediction accuracy before 1000 d, but at 8289 d, a large deviation exists between the predicted and experimental values. The prediction accuracy of the curve obtained by the improved Arrhenius equation is lower than that of the curve obtained by the traditional Arrhenius equation before 3491 d, but after that, the prediction accuracy is improved, and its overall accuracy is higher than that of the traditional Arrhenius equation. Table 4 shows the aging time when the compression deformation retention rate of the sample is reduced to 33.6% and the aging time obtained by the test, respectively, using the traditional Arrhenius equation and the improved Arrhenius equation. It is obvious that the prediction results obtained by the improved Arrhenius equation are closer to the experimental results, and the dispersion coefficient between it and the experimental results is 1.0351, while the dispersion coefficient between the traditional Arrhenius equation prediction results and the experimental results is 1.6653. Therefore, from the long-term aging process, it is feasible to use the improved Arrhenius equation to extrapolate the acceleration factors at lower temperatures, and the accuracy of the result is higher than that predicted through the traditional Arrhenius equation.

## 6. Conclusions

In this study, the artificial bee colony algorithm was used to identify the acceleration factor in the accelerated aging experiment of 8106 ethylene propylene rubber, and the acceleration factor at each aging temperature was coded as a candidate solution, which avoided the error superposition caused by the data fitting at each aging temperature. The acceleration factor obtained by the artificial bee colony algorithm was used to translate the acceleration factor at each aging temperature to the reference temperature, and the sum of squared residuals between the translated data and the decay fitting curve at the reference temperature was 0.043, which proved that the acceleration factor identified by the artificial bee colony algorithm had high accuracy.

Considering the non-Arrhenius phenomenon in the aging process of rubber, the activation energy of rubber is not constant during the aging process, so the power exponent factor is introduced to improve the traditional Arrhenius equation.

The aging life of 8106 ethylene propylene rubber at 25 °C was predicted using the time-temperature equivalent superposition principle based on the acceleration factor obtained from the artificial swarm algorithm identification and the improved Arrhenius equation. The prediction results show that the improved Arrhenius equation has better prediction accuracy for a longer aging process of 8106 ethylene propylene rubber, while the conventional Arrhenius equation has better prediction accuracy for aging processes less than 6 years.

Although the results prove that the artificial bee colony algorithm can provide better results in the aging life prediction of rubber products, the purpose of this paper is not to design a new algorithm that can beat all intelligent algorithms, but to show that the intelligent algorithm is a good alternative in the life prediction of rubber products because it not only simplifies the process of accelerated aging data processing, but also avoids the accumulation of errors in the process, and therefore can provide better results. Therefore, it can provide more accurate prediction results.

## Figures and Tables

**Table 1:** Compression set retention (ε) at different aging temperatures [23].

Temperature	Aging Data								
298.15 K	Aging time (*d*)	71	926	1143	2486	3491	8289		
	ε (%)	96.36	75.89	71.06	66.78	48.26	33.56		
353.15 K	Aging time (*d*)	0.25	1	1.67	3.46	6.71	10.71	16.71	25.71
	ε (%)	82	75.7	70.4	59.9	51.5	46.9	42.5	37.2
363.15 K	Aging time (*d*)	0.42	0.75	1.5	3	5	9	14	25
	ε (%)	75.6	64.7	58.1	49.3	42	35	30.9	22.7
373.15 K	Aging time (*d*)	0.25	0.83	1.58	2.58	4.08	7.08	12.08	21.08
	ε (%)	69.1	56.3	47.1	40.7	36.1	26.7	21	15.7
383.15 K	Aging time (*d*)	0.17	0.5	1	2	4	8	14	
	ε (%)	70.8	50.7	41.4	30.6	25.9	19.9	13.8	

**Table 2:** Identification results of rubber aging acceleration factors at different temperatures.

Temperature (K)	353.15	363.15	373.15	383.15
Acceleration factor	1	1.42	1.86	2.29

**Table 3:** Acceleration factor at 293.15 K obtained through different regression equations.

Regression Equation	$a_{old}$	$a_{new}$
Acceleration factor	0.1625	0.1876

**Table 4:** Comparison of life prediction results between traditional and improved Arrhenius equation.

Value	Traditional Arrhenius Equation	Improved Arrhenius Equation	Experimental Results
Aging time (*d*)	13,804	8580	8289
Dispersion coefficient	1.6653	1.0351	1

## Data Availability

The data presented in this study are available on request from the corresponding author.

## Nomenclature

k.	aging reaction rate
T	aging temperature
A	a temperature-independent constant
a,b	regression coefficients
$E_{a}$	apparent activation energy of the aging reaction
$E_{b}$	equivalent linear activation energy
R	ideal gas constant
n	power factor
α	time-independent constant
a	acceleration factor
$a_{old}$	acceleration factor obtained using the traditional Arrhenius equation
$a_{new}$	acceleration factor obtained using the improved Arrhenius equation
P	aging performance degradation index
y	logarithm of the aging performance degradation index P
${\hat{y}}_{ij}$	estimated value of y
t	aging time
ε	compression set retention
$x_{i}$	vector to be solved for the artificial bee colony algorithm
$x_{i}^{j}$	value of each dimension of $x_{i}$
$v_{i}^{j}$	alternative value of $x_{i}^{j}$ from neighborhood search
Limit	upper limit of the number of searches for a single nectar source by artificial bee colony algorithm
$iter_{\max}$	maximum number of iterations of artificial bee colony algorithm

## Appendix A

**Algorithm A1.** Identifying acceleration factors using an artificial bee colony algorithm.
1: Read the n experimental data values of $\left(t_{ij},P_{ij}\right)$. Then, store them in the matrix; 2: Initialize related parameters of ABC algorithm: $iter_{\max}$ (maximum number of iterations), Limit (maximum number of iterations before abandoning the solution), d (parameter number), N (initial solution number); 3: Initialize the solution vector using Equation (12); 4: Evaluate the fitness value of the initial solution using Equation (14); 5: Set cycle to 1; 6: Repeat; 7: For each employed bee: { Produce new solution $v_{i}$ by Equation (13); Calculate the value $fit_{i}$ by Equation (14); Apply greedy selection process. } 8: Calculate the probability values $P(x_{i})$ for the produced solutions by Equation (15); 9: For each onlooker bee: { Select a solution I depending on $P\left(x_{i}\right)$; Repeat step (7). } 10: If a candidate solution does not change in more than Limit iterations, then replace it with a new random solution produced by a scout bee using Equation (16);
